# Scanning Thermal Microscopy of Ultrathin Films: Numerical Studies Regarding Cantilever Displacement, Thermal Contact Areas, Heat Fluxes, and Heat Distribution

**DOI:** 10.3390/nano11020491

**Published:** 2021-02-16

**Authors:** Christoph Metzke, Fabian Kühnel, Jonas Weber, Günther Benstetter

**Affiliations:** 1 Department of Electrical Engineering and Media Technology, Deggendorf Institute of Technology, Dieter-Görlitz-Platz 1, 94469 Deggendorf, Germany; christoph.metzke@th-deg.de (C.M.); fabian.kuehnel@th-deg.de (F.K.); jonas.weber@th-deg.de (J.W.); 2 Department of Electrical Engineering, Helmut Schmidt University/University of the Federal Armed Forces Hamburg, Holstenhofweg 85, 22043 Hamburg, Germany; 3 Institute of Functional Nano and Soft Materials, Collaborative Innovation Center of Suzhou Nanoscience & Technology, Soochow University, 199 Ren-Ai Road, Suzhou 215123, China; 4 Department of Applied Physics, University of Barcelona, Martí i Franquès 1, 08028 Barcelona, Spain

**Keywords:** scanning thermal microscopy (SThM), numerical study, finite element analysis (FEA), boron nitride, h-BN, ultrathin films, heat transfer, thermal contact, penetration depth, stationary time

## Abstract

New micro- and nanoscale devices require electrically isolating materials with specific thermal properties. One option to characterize these thermal properties is the atomic force microscopy (AFM)-based scanning thermal microscopy (SThM) technique. It enables qualitative mapping of local thermal conductivities of ultrathin films. To fully understand and correctly interpret the results of practical SThM measurements, it is essential to have detailed knowledge about the heat transfer process between the probe and the sample. However, little can be found in the literature so far. Therefore, this work focuses on theoretical SThM studies of ultrathin films with anisotropic thermal properties such as hexagonal boron nitride (h-BN) and compares the results with a bulk silicon (Si) sample. Energy fluxes from the probe to the sample between 0.6 µW and 126.8 µW are found for different cases with a tip radius of approximately 300 nm. A present thermal interface resistance (TIR) between bulk Si and ultrathin h-BN on top can fully suppress a further heat penetration. The time until heat propagation within the sample is stationary is found to be below 1 µs, which may justify higher tip velocities in practical SThM investigations of up to 20 µms^−1^. It is also demonstrated that there is almost no influence of convection and radiation, whereas a possible TIR between probe and sample must be considered.

## 1. Introduction

Since the thermal properties of thin films vary significantly from those of the corresponding bulk materials [1,2,3,4], new promising materials for micro and nanoscale devices require a detailed investigation with advanced techniques. Moreover, due to several atomic and molecular effects, such as grain boundaries, or the transition to ballistic heat transport, the thermal characterization becomes increasingly challenging. Methods for thin-film thermal characterization are also limited by various factors such as film thickness or anisotropic material properties. One possible method is SThM, which is specifically designed to characterize the local thermal properties of thin films. SThM is applied in an AFM system together with additional measurement equipment. It is a method in which a cantilever is in direct physical contact with a sample. The sample is scanned in a special pattern to obtain local thermal properties. SThM thermal images are likely to be influenced by the sample’s topography, which has been explained in literature in recent years [5,6,7,8,9,10,11,12]. To ensure a correct interpretation of the recorded measurement results, a deep understanding of heat transfer during SThM measurements is essential to comprehend the origin and impact of those and further effects. This work aims to provide these insights.

In this work, the goal is to accurately predict the heat transfer process during realistic SThM measurements of ultrathin films such as h-BN and a bulk Si sample. Therefore, theoretical calculations and finite element analysis (FEA) are performed. FEA is a versatile tool to simulate, e.g., heat transfer or mechanical problems that can be described by mathematical equations. Similar SThM measurements with h-BN have been performed in recent works [12]. We tried to create theoretical measurement setups that comply with real scenarios so that the theoretical results may be adopted by other researchers to compare them with practical measurements or to explain certain effects of SThM applied to ultrathin films.

The literature in recent years especially focused on qualitative local thermal properties. Leitgeb, Fladischer et al. investigated 500 nm tungsten films employing SThM based on the 3ω method and compared the results to the time domain thermoreflectance technique. Said tungsten films would have a thermal conductivity between 151.4 Wm^−1^K^−1^ and 156.0 Wm^−1^K^−1^ depending on the heat treatment [13,14]. Chen et al. estimated the thermal conductivity of a single SiO_2_ nanoparticle at 300 K to 0.95 ± 0.08 Wm^−1^K^−1^ using SThM. They also found out that the TIR between the probe and the nanoparticle accounts for 70% of the total thermal resistance. Therefore, TIRs would have to be considered in thermal conductivity measurements [15]. Existing TIRs from metal/non-metal and non-metal/non-metal contacts within a sample were studied by Park et al. using ultrahigh vacuum SThM. They suggest the presented method to be used actively for nanoscale TIR measurements and show the significant contribution of TIRs to the entire heat dissipation at the nanoscale [16]. Chirtoc et al. conclude that the heat management of nanofabricated thermal probes might be optimized by decreasing the TIRs between tip and sample [17]. To obtain local thermal properties using SThM, a tip calibration is necessary. A new calibration method for local temperature measurements is introduced by Nguyen et al., whose active thermal microchips could be used for different SThM probes. In addition, they estimate the TIRs between tip and sample, which would have a great impact on the results [18]. Recent literature demonstrates the necessity of detailed insights into the heat transfer process of SThM measurements, but also the great possibilities of SThM. This work aims to provide values regarding energy fluxes, heat distribution and the influence of possible TIRs between tip and sample. Said values can hardly be found in the literature, especially for h-BN or Si samples, which are interesting in our field of research. We want to support other researchers to fully understand and interpret upcoming effects in their practical SThM investigations.

The measurement and simulation procedure applied in this research work is a sequence of interdependent steps. First, the geometry of a used SThM probe was investigated via SEM (Section 4.1). The information of the SEM images provided the fundamentals to model the probe in SolidWorks (SW). The geometry was then imported into COMSOL Multiphysics (CM). Here, the cantilever displacement under specific forces (Section 4.2) was simulated. After a theoretical calculation of the thermal contact area (TCA) between tip and sample (Section 4.3), we were able to model realistic measurement scenarios using a sample with an ultrathin top surface, which exhibits anisotropic thermal conductivity (Section 4.4). Parametric sweep studies enabled the simulation of different probe temperatures and various anisotropic material properties. Subsequently, these simulations were compared to those with a standard bulk Si sample with isotropic thermal properties (Section 4.5). Both simulation setups deliver particular results concerning the heat distribution and the heat fluxes from tip to sample. Henceforth, the time until the heat transfer process becomes stationary was investigated (Section 4.6). The influence of convection and radiation on the present simulations was also taken into account (Section 4.7), as well as the influence of a possible TIR between tip and sample (Section 4.8). Finally, the applied meshing strategy is verified to demonstrate the reliability of our results (Section 4.9).

## 2. Materials and Methods

AFM is a method to characterize surfaces according to topographical, mechanical, and electrical properties on a nanometer scale. One of the key parts is a microfabricated probe with an ultrasharp tip to contact the sample surface. Tip radii are in the small nm range (depending on the desired application), e.g., Bruker VITA-DM-NANOTA-200 with a tip radius of up to 30 nm and a tip height of 3–6 µm for a new tip [19,20]. The sample is scanned with a predefined number of lines and readings per line, which results in a line-by-line image. SThM is a method to qualitatively map local thermal conductivities and temperatures as a subcategory of AFM that requires additional measuring equipment. In standard applications, SThM measurements are performed in contact mode. Here, the tip is in direct physical contact with the surface under investigation. The force between tip and sample is held constant in most cases and can be defined by the user during the measurement. Typically, the force of contact mode measurements is within the range of 10 nN to 100 nN [21]. Moreover, a thermal signal is measured and assigned simultaneously to the corresponding topography. Thereunto, a thermal resistive probe is heated with a specific heating power. The temperature of the probe depends on the heat exchange between tip and sample and, therefore, on the sample’s local thermal conductivity. If the local thermal conductivity of the sample is high, more heat can spread into the sample, causing a temperature decrease of the probe. The AFM relies on a feedback algorithm and a Wheatstone bridge to evaluate the local thermal conductivities. As a result, SThM measurements create two images simultaneously, one topography and one thermal image of the same position. Such images can be found in [12]. For more details regarding the SThM measurement process, please refer to [22].

In our field of research, special focus is on the thermal characterization of ultrathin films such as h-BN with thicknesses of approximately 10 nm, which are supposed to have anisotropic thermal properties. Recent works demonstrated the possibility of such SThM measurements [12]. This work continues said research and compares it to “standard” SThM measurements of bulk Si with isotropic thermal properties. We deploy the following SThM probe, soft- and hardware:

- Thermal probe: SThM probe Bruker VITA-DM-NANOTA-200 (Bruker Corporation, Billerica, MA, USA) [19,20]; The thermal probe is made from crystalline Si and can be heated repeatedly and reliably up to temperatures of 350 °C, according to manufacturer information. Consecutively, we use the term “probe” for the whole thermal probe (as it can be purchased), the term “cantilever” for the flexible mechanical part of the probe, and the term “tip” only for the sharp area on the front side of the probe, which is in direct contact with the surface of the samples.
- SEM investigation: Zeiss ULTRA 55 (Carl Zeiss AG, Oberkochen, Germany);
- Modeling process: SolidWorks 2020 (SW; Dassault Systemes Deutschland GmbH, Stuttgart, Germany);
- Simulations: COMSOL Multiphysics 5.5 (CM; Comsol Multiphysics GmbH, Göttingen, Germany), which is a versatile FEA tool to simulate, e.g., heat transfer or mechanical problems that can be described by mathematical equations.

## 3. Theoretical Background

### 3.1. Cantilever Displacement

The displacement of a beam w(l), which is fixed at one end and stressed by a single force F at the other end, can be calculated using Equation (1), where l is the cantilever length and E Young’s modulus. The moment of inertia I of a cantilever with a rectangular cross-section can be calculated with $I\;=\;\left(b\cdot h^{3}\right)/12$, in which b is the width and h is the height of the cantilever [23].

(1) $$w\left(l\right)\;=\;\frac{1}{3}\cdot \frac{F\cdot l^{3}}{E\cdot I}\;\mathrm{in}\;\left[m\right]$$

The bending stress σ_b_ can be calculated according to Equation (2) by the division of the bending moment $M_{b}\;=\;F\cdot l$ and the moment of resistance $W\;=\;\left(b\cdot h^{2}\right)/6$ (in case of a rectangular beam) [23].

(2) $$\sigma_{b}\;=\;\frac{M_{b}}{W}\;\mathrm{in}\;\left[{\mathrm{Nm}}^{-2}\right]$$

In Section 4.2, Equations (1) and (2) are used to calculate the cantilever displacement and the bending stress in SThM measurements.

### 3.2. Hertzian Surface Pressure

Hertzian surface pressure occurs when two rigid bodies touch each other. In the special case of a sphere with radius R, Poisson’s ratio ν_1_ and Young’s modulus $E_{1}$ touching a flat surface with Poisson’s ratio ν_2_ and Young’s modulus $E_{2}$ under a force F, the indentation depth d can be calculated using Equation (3) [24].

(3) $$d\;=\;\sqrt[{3}]{{\left(\frac{3F}{4E_{\prime }\cdot \sqrt{R}}\right)}^{2}}\;\mathrm{in}\;\left[m\right]$$

E’ is the combined Young’s modulus and can be calculated according to Equation (4) [24].

(4) $$E_{\prime }=\;\frac{E_{1}\cdot E_{2}}{\left(1-\nu_{1}^{2}\right)\cdot E_{2}+\left(1-\nu_{2}^{2}\right)\cdot E_{1}}\;\mathrm{in}\;\left[{\mathrm{Nm}}^{-2}\right]$$

The touching radius r can then be calculated with $r\;=\;\sqrt{R\cdot d}$ [24]. It must be stated that using the touching radius r does not lead to the mathematical exact contact area as the contact area is not a flat circle but a small section of a sphere. However, the calculated indentation depths are by far smaller than the touching radius ($d\left(r\right)\;={\;r}^{2}/R$; e.g., d = 0.1 nm and r = 5.7 nm with r_tip_ = 300 nm and tip force = 100 nN) and can thus be neglected. We, therefore, use the touching radius r for the calculation of the TCAs in SThM measurements (Section 4.3) as an adequate approximation for the simulations in Section 4.4 to Section 4.9.

### 3.3. Heat Transfer

Equation (5) represents the general heat conduction equation in the three-dimensional case without inner heat sources, in which the quotient k/(ρ⋅c) is expressed by the thermal diffusivity a [25].

(5) $$\frac{\mathrm{dT}}{\mathrm{dt}}\;=\;\frac{k}{\rho \cdot c}\cdot \left(\frac{\partial^{2}T}{\partial x^{2}}+\frac{\partial^{2}T}{\partial y^{2}}+\frac{\partial^{2}T}{\partial z^{2}}\right)\;=\;a\cdot \Delta T$$

If there are inner heat sources h(t) (e.g., joule heating or chemical reactions), the inhomogeneous heat conduction Equation (6) follows (5) [25]. In this work, thermal conduction is applied to the entire geometry of the simulations in Section 4.4 to Section 4.9. CM solves said equations using numerical methods.

(6) $$\frac{\mathrm{dT}}{\mathrm{dt}}\;=\;a\cdot \Delta T+h\left(t\right)$$

Convection is a mass bound energy transport caused by macroscopic particle movement. Considering free convection, this flow mainly results from the temperature-dependent particle movement and the thermal buoyancy. The reason for this is that fluids normally expand with increasing temperatures and therefore exhibit lower densities. Forced convection occurs if, e.g., a ventilator causes the flow field. Forced convection then overlaps with free convection. The influence of free convection is studied in Section 4.7 using Equation (7), where α is the heat transfer coefficient, which depends on the materials, surfaces and ambient conditions, $A_{\mathrm{conv}}$ the convective area and $T_{2}$ and $T_{1}$ the temperatures of the material and the surrounding fluid (e.g., air), respectively [25]. In this work, the influence of convection can be neglected. This is further discussed in Section 4.7.

(7) $${\;\dot{Q}}_{\mathrm{conv}}\;=\;\alpha \cdot A_{\mathrm{conv}}\cdot \left(T_{2}-T_{1}\right)\;\mathrm{in}\;\left[W\right]$$

Thermal radiation is not mass bound. Therefore, it occurs even under high vacuum conditions. Electromagnetic waves run from one body surface to another. Each body absorbs and emits radiation. In the special case of a small body with an area $A_{\mathrm{rad}}$ and temperature $T_{2}$ surrounded by a much greater emissive area with a temperature $T_{1}$, the net radiative heat flow of the body can be calculated with Equation (8), using the Stefan-Boltzmann law [25].

(8) $${\;\dot{Q}}_{\mathrm{rad}}\;=\;-\epsilon_{\mathrm{rad}}\cdot \sigma \cdot A_{\mathrm{rad}}\cdot \left(T_{2}^{4}-T_{1}^{4}\right)\;\mathrm{in}\;\left[W\right]$$

The negative radiation constant $-\epsilon_{\mathrm{rad}}$ depends on the considered body, and σ is the Stefan-Boltzmann constant. When $T_{2}$ is greater than $T_{1}$ the sign of ${\;\dot{Q}}_{\mathrm{rad}}$ is negative which means, that the considered body loses energy. In this work, the influence of radiation of the heated SThM cantilever can be neglected. This is further discussed in Section 4.7.

## 4. Results and Discussion

### 4.1. SEM Investigation of a Used SThM Tip

**Figure 1:**
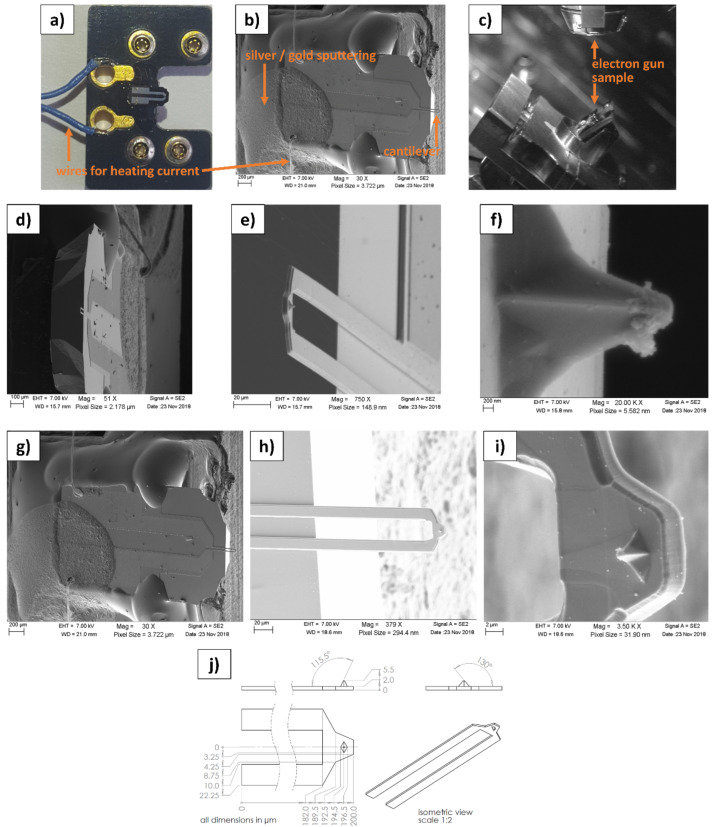
(**a**) Photography image of the scanning thermal microscopy (SThM) probe Bruker VITA-DM-NANOTA-200 before preparation for SEM. (**b**) SEM image of a fully prepared tip. The sputtering area, wires for the heating current, and the cantilever are visible. (**c**) Image of the sample fixation inside the SEM. (**d**) Side-view close-up of the tip in (**e**,**f**). (**g**) Top-view close-up of the tip in (**h**,**i**). (**j**) Dimensions of the probe used for the simulations and isometric view. This SEM investigation made it possible to estimate the geometry of the cantilever and the radius of the used tip, which is approximately 300 nm.

To obtain vital information about the geometry of the cantilever and tip, we performed an SEM investigation of the thermal SThM probe Bruker VITA-DM-NANOTA-200 [20], which was already in use. This facilitated modeling the probe in detail in SW and paved the way for the simulations. SEM investigations require a conductive connection between the sample and the holder to avoid electrical overcharging caused by trapped electrons from the electron beam. Therefore, the probe was fixed on a sample holder using conductive silver and gold in a sputtering process. Figure 1a–c shows the probe before and after the preparation process, as well as inside the sample chamber of the SEM. To collect precise geometry data, the probe was investigated in two positions, one side view and one top view. The results are depicted in Figure 1d–i. Therefore, the tip radius was estimated to be approximately 300 nm. The geometry data for the simulations were created upon these images and are depicted in Figure 1j.

We assume a tip radius of 300 nm to represent an “average” used probe. However, an “average” used probe can hardly be defined as it depends on various factors such as the number of measurements, tip velocities and forces, sample materials, and many more. Mostly, SThM probes are used as long as they deliver reliable results, and from our experience, such tips exhibit tip radii within the region of 300 nm. Furthermore, new tips may degrade much faster than used tips, which is the reason why larger tip radii of around 300 nm may occur more often in practical SThM measurements. As the main residue is not located directly at the TCA (see Figure 1f), we assume it to have no influence. However, residues may increase the TIR between tip and sample. This effect is further discussed in Section 4.8.

### 4.2. Cantilever Displacement and Von Mises Stress

To study the displacement and the von Mises stress of the cantilever, a 3D-simulation was set up. The cantilever (geometry see Figure 1j) is fixed by the two connectors on one end. The simulated load is applied directly to the cantilever tip on the other end. All other edges and areas are set to be free. Moreover, gravity is set to a value of 9.81 ms^−2^ in the z-direction. For the cantilever, the material bulk Si was used with Young’s modulus of 131 GPa and a Poisson’s ratio of 0.221 [26]. The mesh was built with the option *Physics-controlled extremely fine*, which provides the finest automatically built mesh in CM. The study was performed in the stationary regime.

**Figure 2:**
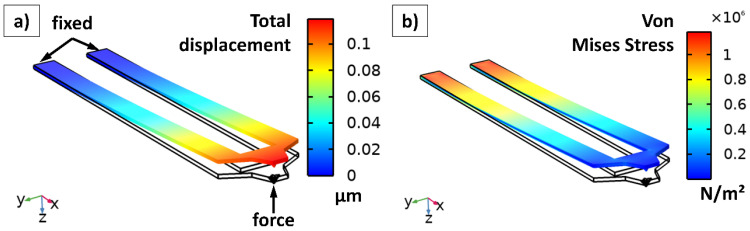
Simulation results under a tip force of 100 nN. (**a**) Total displacement in µm. (**b**) Von Mises stress in Nm^−2^.

Figure 2a illustrates the total displacement in µm, (b) represents the von Mises stress in Nm^−2^ under a tip force of 100 nN, which acts directed towards the tip in the negative z-direction. Logically, the total displacement reaches its maximum value at the largest x-coordinate, whereas the von Mises stress achieves its maximum directly at the clamping. Here are some important simulation results for a tip force of 100 nN. The simulation results for 10 nN and 1000 nN can be derived by the multiplication of the following values with 0.1 and 10, respectively:

- Total displacement: 0.114 µm at the center point of the tip and 0.119 µm at the free end of the cantilever;
- Maximum von Mises stress at the fixed end of the cantilever: 1.26 × 10^6^ Nm^−2^.

These values were also calculated manually for a simplified cantilever with a rectangular cross-section (b = 24.5 µm, h = 2 µm and l = 194.5 µm) fixed on only one end using the Equations (1) and (2). The results of the calculations are w (l = 194.5 µm) = 0.115 µm and σ_b_ = 1.19 × 10^6^ Nm^−2^. Compared to the simulation results above (0.114 µm and 1.26 × 10^6^ Nm^−2^), it is obvious that the exact geometry around the tip has just little influence on the displacement and the maximum von Mises stress. The breaking strength of Si is assumed to be in the range from 5 × 10^7^ Nm^−2^ to 20 × 10^7^ Nm^−2^ [27]. Compared to the maximum von Mises stress at the fixed end of the cantilever (1.26 × 10^6^ Nm^−2^), there is a safety factor of approximately 40 to 160 before the cantilever breaks. Based on this calculation, the maximum tip forces that can be applied to the tip without breaking the cantilever are in the range from 4 µN to 16 µN. However, such values will not be reached in reasonable SThM measurements.

### 4.3. Calculation of TCAs in Realistic SThM Investigations

**Figure 3:**
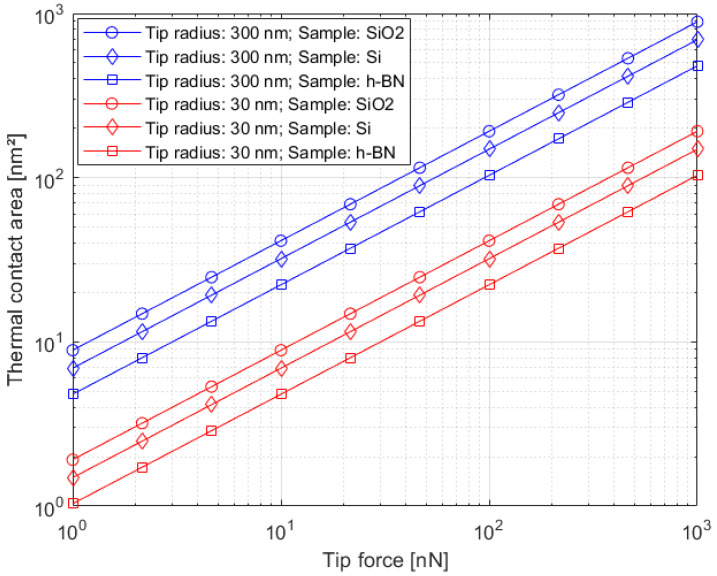
Calculated TCAs in realistic SThM measurements for different probe-sample combinations. The red curves represent a new probe with a tip radius of 30 nm, and the blue curves represent an “average” used probe with a tip radius of 300 nm. A similar investigation can be found in [30].

To estimate TCAs between an SThM probe and different samples, we performed a mathematical evaluation regarding Equations (3) and (4). We calculated six different cases: a new Si probe with a tip radius of 30 nm and an “average” used to probe with a tip radius of 300 nm each combined with the Si sample, SiO_2_ sample and h-BN sample. The tip radius (300 nm) for an “average” used probe was determined by SEM investigations (Section 4.1). For the calculations, we used the material properties in Table 1. The calculated thermal contact areas are depicted in Figure 3.

We chose the above described tip-sample combinations for reasons of reusability as these configurations are comparatively common and might also occur in similar SThM investigations of other researchers. We also used the calculated TCAs of the h-BN curves to calculate the thermal contact radius (TCR) for the simulations in Section 4.4 to Section 4.9.

Appropriate material properties for h-BN are not easy to estimate, as in ultrathin (2D) materials, they are also strongly dependent on factors such as material orientation and exact film thickness. However, in our calculations concerning the h-BN sample, the Poisson’s ratio has just little influence on the calculated contact area. The main factor is Young’s modulus, as it is assumed to be extremely high in comparison to Si and SiO_2_. Consequently, if other researchers might find different values of Young’s modulus of h-BN appropriate for their individual case, the contact areas can be approximated to lie in between the h-BN and SiO_2_ curves in Figure 3. Prerequisites are a similar measurement setup and Young’s modulus between 70 and 850 GPa. Nevertheless, it must be stated that the lower Young’s modulus, the higher becomes the influence of different values for the Poisson’s ratio.

For our simulations, we assume the calculated ideal contact areas in this section to be realistic, as the impact of surface roughness decreases with lower surface roughnesses. We also assume roughness to be neglectable as we work with samples exhibiting surface roughnesses in the sub-nm area. In the following sections, we use the calculated TCAs to simulate the heat transfer in SThM measurements.

### 4.4. Heat Spreading in SThM Measurements Regarding Ultrathin h-BN Film

To study the heat spreading in SThM measurements on a sample with an ultrathin h-BN film on top, this simulation was set up.

**Simulation setup:** For the cantilever (dimensions see Figure 1j), the CM-predefined material bulk Si was set. The rectangular sample (footprint 100 µm × 100 µm) consists of the CM-predefined material h-BN with a thickness of 10 nm on top of 10 µm thick bulk Si. Regarding the anisotropic thermal properties of h-BN, we assume a cross-plane thermal conductivity of h-BN of 1 Wm^−1^K^−1^. The in-plane thermal conductivity of h-BN was defined using the parameter k, which varies depending on the simulations [31]. The thermal contact resistance between h-BN and bulk Si is assumed to be 4 × 10^−8^ Km^2^W^−1^. However, this value can only be estimated and will vary in practical measurements depending on material quality, ambient conditions and manufacturing processes. We chose this value inspired by investigations on similar material stacks such as graphene/Si [32] since the atomic structure of graphene is similar to h-BN. Villaroman et al. estimate the thermal contact resistance between graphene and Si to 3.1–4.9 × 10^−8^ Km^2^W^−1^ [32]. Values in the same order of magnitude can be found in [33,34].

**Figure 4:**
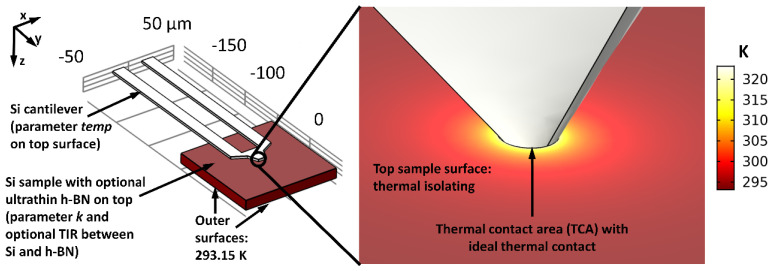
Simulation setup and boundary conditions. Left: The general setup consists of the heated SThM probe and the sample. Right: zoom into the TCA with ideal thermal contact between tip and sample.

The initial value for the temperature was set to 293.15 K for all boundaries. Between tip and sample, an ideal thermal contact was defined. We calculated the TCR of the TCA as follows: With Figure 3 (blue squared h-BN curve), we assumed a realistic TCA of ~103.4 nm^2^ for r_tip_ = 300 nm and a tip force of 100 nN, which leads to a TCR of ~5.7 nm ($\mathrm{TCA}={\mathrm{TCR}}^{2}\cdot \Pi$). This appears to be a realistic value for an ideal thermal contact between a used SThM probe and a sample with an ultrathin h-BN film on top. The temperature of the topside of the cantilever is defined using the parameter *temp*, which varies between 50 °C and 200 °C depending on the simulations. These are appropriate cantilever temperatures for practical SThM investigations [12]. The remaining outer areas of the cantilever were defined as thermal isolating as well as the top surface of the sample, while the remaining outer areas of the sample take over the ambient temperature of exactly 293.15 K. An overview of the simulation setup and boundary conditions is given in Figure 4. The center point of the TCA defines the origin of the coordinate system.

**Figure 5:**
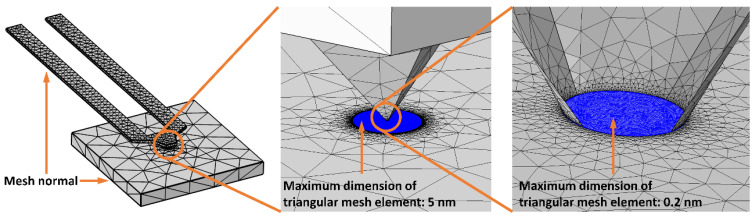
Meshing strategy zooming from the geometry overview to the small TCA.

**Meshing strategy:** To simulate the thermal contact with sufficient resolution, a user-controlled mesh was created. We defined the maximum dimension of a triangular mesh element for the smallest area of the entire simulation, the TCA of the cantilever, to 0.2 nm. Moreover, a circle with a radius of 500 nm around the TCA was defined, in which the maximum dimension of a mesh element is 5 nm. For the remaining geometry of the cantilever and sample, we used the predefined meshing strategy with element size normal. In the present simulations, this meshing strategy represents a good tradeoff between accuracy and simulation time and delivers trustworthy results. The meshing strategy is evaluated in Section 4.9, in which the reliability of our results is demonstrated. Figure 5 illustrates the applied meshing strategy in more detail.

**Parametric studies:** To investigate the cross- and in-plane heat distribution for different cases, a parametric sweep study in the stationary regime was performed. Every single combination of the parameters *k* and *temp* was calculated, which are defined as follows:

- *k*: Ratio between in-plane and cross-plane thermal conductivity $k\;={\;\lambda }_{\|}/\lambda_{⊥}\;$with $\lambda_{⊥}\;=\;1{\;\mathrm{Wm}}^{-1}K^{-1}$ [31]. In the parametric sweep studies in this section, *k* resembles the values 1, 2, 5, 10, 20, 50, and 100;
- *temp*: Temperature of the top surface of the SThM cantilever (boundary condition in Figure 4). In the parametric sweeps, *temp* takes on the values from 50 °C to 200 °C in steps of 25 °C. For reasons of better differentiation and easier comparison with practical measurements, *temp* is always specified in °C, whereas all other temperatures are specified in K.

**Simplifications:** Simulations can never represent the real world as the results are only as good as the chosen simulation model. We tried to create the simulation models as realistic as possible. However, with regards to simulation time and the reusability of our results, we also had to introduce simplifications. The chosen simplifications do not decisively influence our results and are therefore justified. The main simplifications are listed below:

- We assume an ideal thermal contact between tip and sample in Section 4.4 to Section 4.7. TIRs in practical SThM measurements are quite hard to estimate as they depend on numerous factors such as surface roughness, material combination, vertical steps, tip material and geometry, contaminations, tip force, and some more. Due to these numerous influences, TIR will also vary greatly during a single SThM measurement. In literature, values in the range of 10^−8^ Km^2^W^−1^ to 10^−10^ Km^2^W^−1^ for different probes, samples, and measurement scenarios can be found [35,36,37]. Indeed, we assume the TIR to vary in a broader range due to the great number of possible measurement scenarios. To realize a comparison between the simulations in Section 4.4 and Section 4.5, we assume an ideal thermal contact in said sections. The influence of a possible TIR is further studied in Section 4.8. It must also be stated that the present investigation in Section 4.4 focuses on ultrathin h-BN samples, which in fact are super flat as they are built of a stack of single h-BN layers. Roughnesses of high-quality h-BN are assumed to be less than 0.4 nm [38], which is significantly lower compared to a tip radius of 300 nm. As surface roughness has a great impact on TIR, values for h-BN samples should be lower compared to samples with higher roughnesses;
- We neglect a possible water meniscus around the tip. Other researchers also propose that a water meniscus can often be neglected in SThM measurements [22]. On one hand, a present water drop could cause heat conduction and may increase the amount of heat flux between tip and sample slightly. On the other hand, SThM measurements can also be performed under high vacuum conditions, where the appearance of a water meniscus can be excluded. We, therefore, focus on simulations without a possible water meniscus;
- In practical SThM measurements, the probe is scanning over the surface with a specific velocity. This is not presentable in simulations in the stationary regime, where a motionless probe is assumed. However, we justify such simulations in Section 4.6 through the investigation of the time until heat distribution is stationary;
- We also neglect radiation and convection as the influence on our simulated cases is neglectable. This is further discussed in Section 4.7.

**Figure 6:**
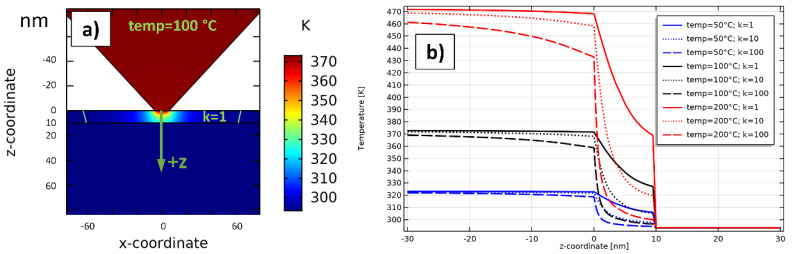
Simulation regarding ultrathin (10 nm) h-BN film on top of Si. (**a**) Stationary heat distribution for *k* = 1 and *temp* = 100 °C on the x/z-plane through the center of TCA (y = 0). (**b**) Temperature curves along the z-coordinate through the center point of TCA for various simulated cases.

**Figure 7:**
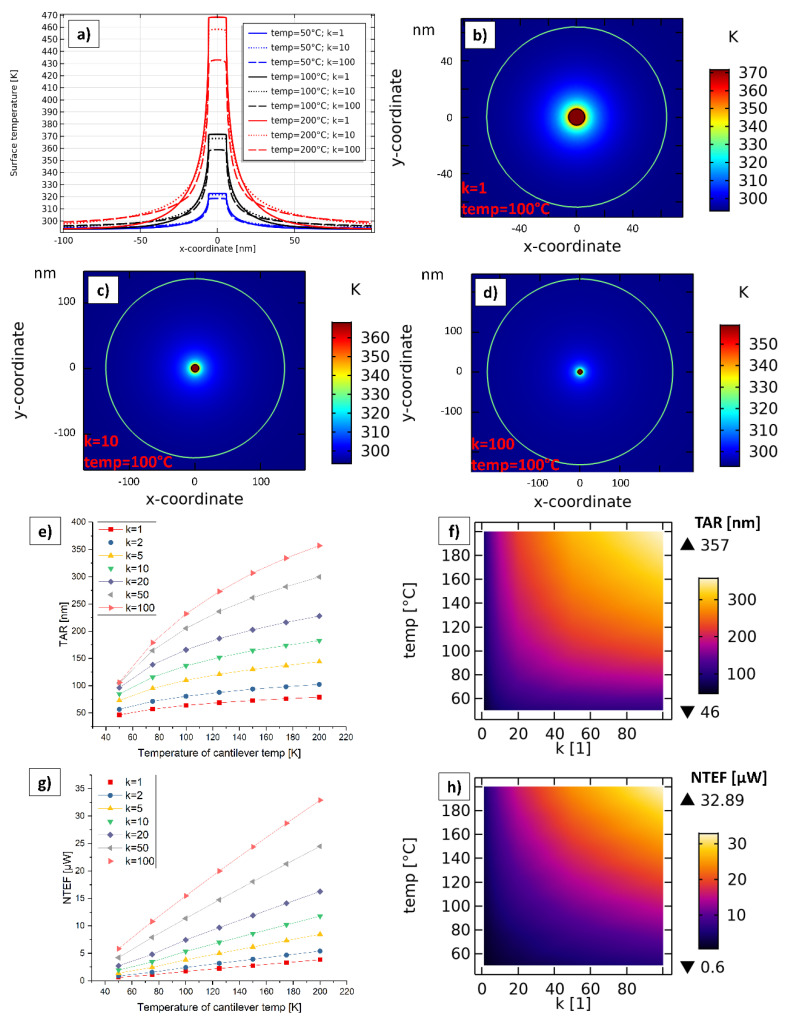
Simulation regarding ultrathin (10 nm) hexagonal boron nitride (h-BN) film on top of Si. (**a**) Temperature curves along the x-coordinate on the top surface (y = z = 0) for different simulated cases. (**b**) to (**d**) Heat distribution and thermal active radius (TAR) on x/y-plane for *temp* = 100 °C and *k* = 1 in (**b**), *k* = 10 in (**c**) and *k* = 100 in (**d**). (**e**) TAR in dependency of *temp* and *k* for all simulated cases. (**f**) Interpolated color plot of the simulated TARs in (**e**). (**g**) normal total energy flux (NTEF) through the TCA in dependency of *temp* and *k* for all simulated cases. (**h**) Interpolated color plot of the simulated NTEFs in (**g**).

**Results:** To compare single measurements, it is necessary to define useful measurable parameters. Hence, we define the thermal active radius (TAR) located on the top surface of the sample starting at the geometric center point of the tip. The temperature until the TAR is greater than 294.15 K, which represents a temperature rise of 1 K in comparison to ambient conditions. In Figure 6b, the heat flow in the z-direction is investigated. It is obvious that with increasing *temp*, the temperature at TCA also increases. However, an increasing *k* results in a greater heat spreading in x and y-direction, which leads to a stronger temperature decrease within the ultrathin h-BN film and the tip (compare curves of the same color each in Figure 6b. It can also be observed that the heat penetration in z-direction ends at the z-coordinate 10 nm, which is exactly where the h-BN ends. The thermal contact resistance between h-BN and Si causes this rapid temperature decrease. Thus, no heat is transferred into the bulk Si. The green curve in Figure 6a illustrates the isothermal line of the TAR. With increasing *k* and *temp,* this line moves to greater values of x, which can also be seen in Figure 7b–d.

Figure 7a illustrates the heat distribution along the x-direction (y = z = 0). It can be seen that higher values of *k* result in a greater TAR. This can also be seen in Figure 7b–d for the special case *temp* = 100 °C. With an increasing *k,* the green isothermal circle line of the TAR moves to greater values (64 nm @ *k* = 1 and 232 nm @ *k* = 100), indicating that heat spreading on the x/y-plane of h-BN increases. Furthermore, we can see that the heat spreading effect between *k* = 1 and *k* = 10 is greater than between *k* = 10 and *k* = 100 (compare curves of the same color in Figure 7a. There seems to be a kind of saturation effect for an increasing *k*. Surely, the temperature of TCA increases with increasing *temp* (Figure 7a). A higher *k* leads to an effective greater thermal conductivity of h-BN. The small TCA then has an increased proportion of the entire thermal resistance, which is the reason for lower temperatures at the TCA with *temp* = const. and *k* increasing.

Figure 7e shows the simulated values for the TAR as a graphical representation. The TAR increases with an increasing *temp,* but there seems to be a kind of saturation effect. The TAR logically also increases with higher *k* as heat spreading is greater there. Values for the TAR lie in between 46 nm (@ *temp* = 50 °C and *k* = 1) and 357 nm (@ *temp* = 100 °C and *k* = 100). Figure 7f delivers a color plot of the TARs in dependency of *k* (x-axes) and *temp* (*y*-axes). This color plot was created using the simulated values in Figure 7e). Intermediate points are interpolated. Depending on the case, other researchers can estimate the TAR for their research using Figure 7a–f. The thermal penetration depths for all cases is exactly 10 nm.

We also simulated the normal total energy flux (NTEF) in the stationary state, which flows through the small TCA for every case. Figure 7g illustrates the simulated cases. The NTEF seems to increase nearly linearly for an increasing *temp*. The NTEF also increases with greater *k*. Values for the NTEF vary from 0.6 µW (@ *temp* = 50 °C and *k* = 1) to 32.9 µW (@ *temp* = 100 °C and *k* = 100). Figure 7h shows a color plot of the NTEF in dependency of *k* (x-axes) and *temp* (y-axes). This color plot was created using the simulated values in Figure 7g. Intermediate points are interpolated. It, therefore, allows a rough estimation for other research for different cases.

A comparison of the NTEFs to measured values of other researchers is quite hard. There is an almost infinite number of possible measurement setups, and our specific setup could not be found in literature so far, to the best of our knowledge. However, the following references used different setups but may verify our simulated range of the NTEFs. Hwang et al. measured heat fluxes during null-point SThM in the range of approximately 1 µW (Teflon-coated surface) and 6 µW (SiO_2_ surface) using a thermocouple probe [39]. Assy and Gomès used a Wollaston wire probe at 140 °C and a Kelvin nanotechnology probe at 65 °C on germanium and Si samples. They calculated the probe Joule power relative difference ∆P/P = (P_c_ − P_a_)/P_c_, where P_c_ and P_a_ represent the probe Joule power in contact and out of contact, respectively. Unfortunately, only relative values of ∆P/P ranging from 0.003 to 0.058 are presented [40].

### 4.5. Heat Spreading in SThM Measurements Regarding a Bulk Si Sample

**Figure 8:**
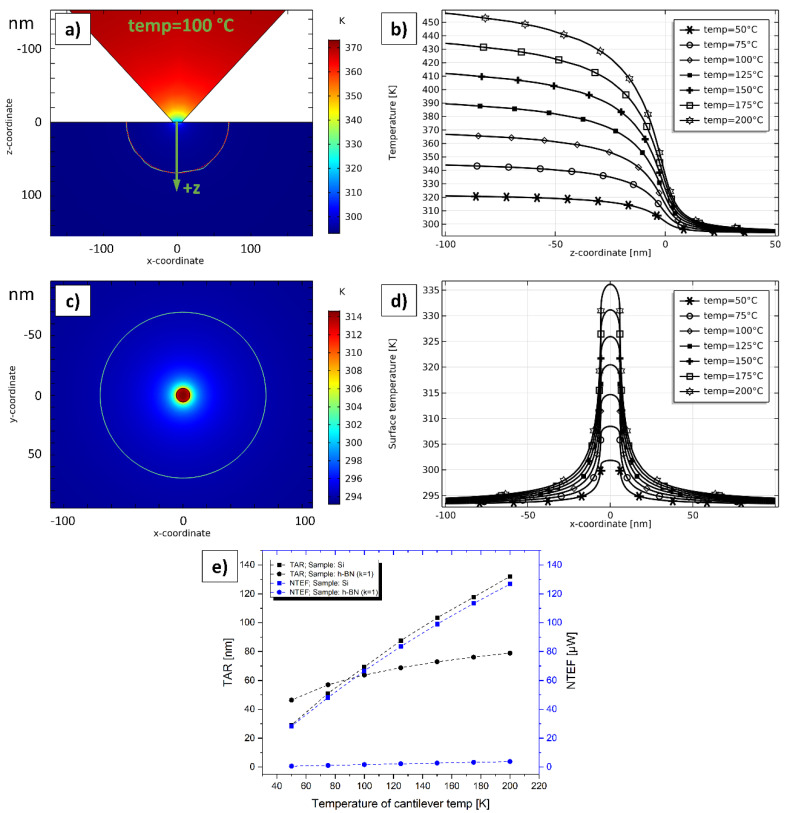
Simulation regarding bulk Si sample. (**a**) Stationary heat distribution for *temp* = 100 °C on the x/z-plane through the center of the TCA (y = 0). (**b**) Temperature curves along the z-coordinate through the center point of the TCA for different simulated cases. (**c**) Heat distribution and TAR on x/y-plane for *temp* = 100 °C. (**d**) Temperature curves along the x-coordinate on the top surface (y = z = 0) for different simulated cases. (**e**) TAR and NTEF in dependency of *temp* for all simulated cases of the Si sample and comparison to simulations with the h-BN sample (isotropic case with *k* = 1).

To compare the results of Section 4.4 to a realistic SThM measurement with a bulk Si sample, this simulation was set up. The only difference to the simulation in Section 4.4 is that the 10 nm thick h-BN layer is removed. The main results are presented in Figure 8.

Figure 8a shows the heat distribution for *temp* = 100 °C on the x/z-plane, whereas (c) shows the same heat distribution on the x/y-plane. Since the Si sample features isotropic thermal conductivity, the heat spreading is more or less circular around the tip. The green line is the TAR-line, where the temperature rise regarding the ambient temperature of 293.15 K is 1 K. As a reason of the mesh density, this line is not exactly a semicircle. The ideal semicircle is represented by the red dotted line, which overlaps the green semicircle. It can be seen that there is only a little deviation from the ideal semicircle caused by the mesh density. Compared to the h-BN sample (Section 4.4), we can see that the penetration depth is not limited by a TCR at 10 nm, and therefore, the penetration depth is much larger than for the h-BN sample. Figure 8b illustrates the temperature drop in z-direction exactly through the center of the TCA for all simulated cases. It is obvious that with increasing *temp,* the temperature at the TCA and in general also increases. Temperatures at z = 0 nm, which is directly at the physical center point of the TCA, are in between 301.8 K (@ *temp* = 50 °C) and 336.1 K (@ *temp* = 200 °C). Compared to the h-BN sample, we can see that the temperature drop within the cantilever is much higher. This is because Si has a higher overall thermal conductivity than h-BN, whereby the thermal resistance of the small TCA creates a greater proportion of the entire thermal resistance. Figure 8d shows the temperature curves along the x-direction (y = z = 0). As expected, with an increasing *temp* the curves also rise.

Figure 8e illustrates the TAR and the NTEF in dependency of *temp* for all simulated cases of the Si sample and compares them to the simulations of the isotropic case (*k* = 1) of the h-BN sample in Section 4.4. It is obvious that the NTEFs are significantly greater for the Si sample and increase with increasing *temp*. In general, the NTEFs for the Si sample are in between 28.3 µW (@ *temp* = 50 °C) and 126.8 µW (@ *temp* = 200 °C), the TARs are ranging from 29 nm (@ *temp* = 50 °C) to 132 nm (@ *temp* = 200 °C). The TARs of the Si sample are greater compared to the h-BN sample from *temp* ≈ 90 °C upwards. Values for the NTEF are also significantly greater compared to the maximum curves of the h-BN sample with *k* = 100 in Figure 7g. The root cause for this is the greater overall thermal conductivity of Si.

### 4.6. Stationary Time of Heat Distribution

**Figure 9:**
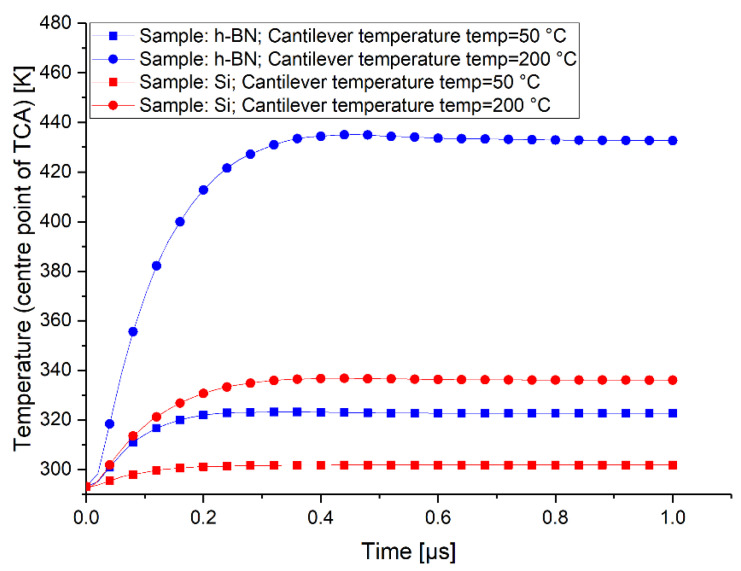
Time-dependent simulation of the temperature rise at the center point of the TCA for the minimum and maximum cases of the h-BN and Si sample, respectively.

To study the time until heat dissipation is stationary (t_stat_), we performed the subsequent simulations. The simulations are based on Section 4.4 and Section 4.5, with the difference being time-dependent instead of stationary. We consider the minimum and maximum case regarding the NTEF through the TCA for both the h-BN and the Si sample. We consider the time-dependent temperature curve for the geometric center point of the TCA. Those four curves are depicted in Figure 9.

As a result, we can say that the t_stat_ for every simulated case is below 1 µs. In general, it seems that higher cantilever temperatures lead to a slightly greater t_stat_. In practical SThM measurements, the cantilever is usually moving over the sample with a specific velocity. This is not representable in simulations. The tip velocity in practical SThM measurements is normally below 20 µms^−1^ [12], which means that in 1 µs, the tip moves less than 20 pm. This is by far smaller than the atomic radius of Si or the lattice constant of h-BN. Therefore, simulations in the stationary regime in Section 4.4 and Section 4.5, which assume a motionless cantilever, are justified.

### 4.7. Influence of Convection and Radiation

To study the influence of radiation, we set up the same simulation as in Section 4.5, with the only difference of surface radiation being enabled for all areas which were isolated in Section 4.5. Surface emissivity $\epsilon_{\mathrm{rad}}$ of Si was set to 0.67 [41]. We also consider the minimum and maximum cases regarding the NTEF. By comparing the NTEF and the TAR for the minimum and maximum cases, we obtain the same results whether radiation being enabled or disabled (28.3 µW and 29 nm @ *temp* = 50 °C; 126.8 µW and 132 nm @ *temp* = 200 °C; compare also Figure 8e). Therefore, we can say that the influence of radiation can be neglected in our simulations. To roughly estimate the amount of radiative heat losses, a radiative area $A_{\mathrm{rad}}$ with a radius of 100 nm and a constant surface temperature $T_{2}$ of 393.15 K with an ambient temperature $T_{1}$ of 293.15 K and an $\epsilon_{\mathrm{rad}}$ of 0.67 is considered. Using Equation (8), we calculate radiative heat losses of ~19.7 pW.

To roughly estimate the influence of convection a manual calculation with a convective heat transfer coefficient α of −5 Wm^−2^ K^−1^ [42], a convective area $A_{\mathrm{conv}}$ with a radius of 100 nm, a constant surface temperature $T_{2}$ of 393.15 K and an ambient temperature $T_{1}$ of 293.15 K is considered. Using Equation (7), we reach a loss of power caused by free convection of ~15.7 pW.

Finally, we may state that estimated convective and radiative heat losses in this work are extremely low compared to the simulated NTEF in Figure 7g and Figure 8e. A comparative simulation also shows no differences regarding the NTEF and the TAR with radiation being enabled or disabled. Therefore, we neglect the influence of convection and radiation in our simulations. Furthermore, in the literature, the influence of radiation and convection is neglected in special cases of SThM measurements [22].

### 4.8. Influence of the TIR at the TCA regarding the TAR and the NTEF

**Figure 10:**
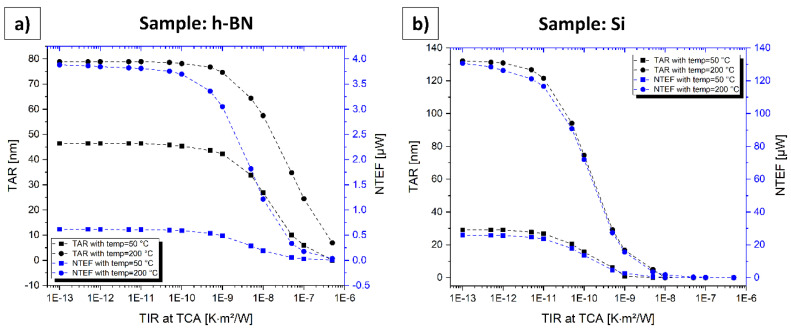
TAR and NTEF in dependency of the TIR at the TCA. (**a**) Sample: h-BN (compare Section 4.4). (**b**) Sample: Si (compare Section 4.5).

To study the influence of a possible TIR between probe and sample directly at the TCA, these simulations were set up. TIRs in practical SThM measurements are quite hard to estimate as they depend on numerous factors such as surface roughness, material combination, vertical steps, tip material and geometry, contaminations, residue, tip force, and some more. Due to these numerous influences, the TIR will also vary significantly during a single SThM measurement. In literature, values in the range of 10^−8^ Km^2^W^−1^ to 10^−10^ Km^2^W^−1^ for different probes, samples, and measurement cases can be found [35,36,37]. Indeed, we assume the TIR to vary in a broader range due to the great number of possible measurement scenarios. To enable a comparison of the simulations in Section 4.4 and Section 4.5, we assume an ideal thermal contact in said sections. In contrast, here, the influence of a varying TIR is investigated. Figure 10 illustrates the simulation results. We created the same simulations as in Section 4.4 (with *k* = 1) and Section 4.5 for *temp* = 50 °C and *temp* = 200 °C with the difference of varying the TIR from 5 × 10^−7^ Km^2^W^−1^ to 1 × 10^−13^ Km^2^W^−1^.

The case with an ultrathin h-BN film on the top surface is demonstrated in Figure 10a, representing ultrathin films with low thermal conductivities. It can be deduced that both, the TAR and the NTEF increase with decreasing TIR and converge exactly at the same values for the corresponding case in Section 4.4 (46 nm and 0.6 µW @ *temp* = 50 °C; 79 nm and 3.8 µW @ *temp* = 200 °C). This convergence starts approximately from 1 × 10^−10^ Km^2^W^−1^ downwards. Figure 10b represents the case of the bulk Si sample as a sample with high thermal conductivity. Values for the TAR and the NTEF also converge at similar values compared to the corresponding cases in Section 4.5 (29 nm and 28.3 µW @ *temp* = 50 °C; 132 nm and 126.8 µW @ *temp* = 200 °C). Compared to the h-BN sample, this convergence effect starts with significantly lower TIR values at approximately 1 × 10^−12^ Km^2^W^−1^. However, it seems that in samples with a higher thermal conductivity, the influence of the TIR also increases. Nevertheless, it must be stated that SThM measurements with different material combinations cannot be compared directly, as the TIR depends on numerous influence factors as listed above. As a result, we may conclude that a TIR at the TCA has some influence and will reduce the ideal simulated values of the TAR and the NTEF in Section 4.4 and Section 4.5. This effect should be considered by other researchers assuming specific values for the TIR.

### 4.9. Mesh Verification

**Figure 11:**
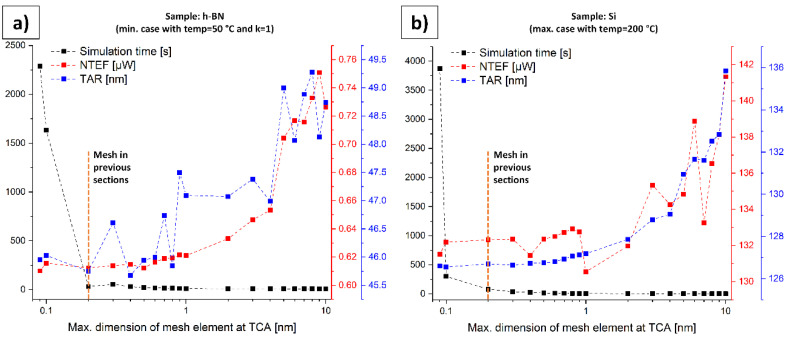
Dependency of the NTEFs and the TARs on the mesh density and confrontation with the simulation time. (**a**) Sample: h-BN (compare Section 4.4). (**b**) Sample: Si (compare Section 4.5).

To demonstrate the reliability of the results, we set up two parametric simulations. They are based on the simulations in Section 4.4 (h-BN sample) and Section 4.5 (Si sample). For the h-BN sample, the minimum case regarding the NTEF with *temp* = 50 °C and *k* = 1 was simulated, whereas for the Si sample, the maximum case regarding the NTEF with *temp* = 200 °C was performed. The sweep parameter in these simulations is the maximum size of a triangular mesh element within the TCA (see Figure 5 right). This parameter was swept between 0.09 nm and 10 nm to obtain information about the dependency of the NTEFs and the TARs on the mesh density and to confront them with the simulation time. Additionally, the maximum size in the circle around the TCA (see Figure 5 center) is defined to be 25 times larger than the maximum size within the TCA. Figure 11 illustrates the findings.

It is obvious that with a finer mesh (left part of the *x*-axis), the values for the NTEFs and the TARs show a kind of saturation effect. For the h-BN sample, there is only little change for x values smaller than 0.2 nm, which was the chosen mesh density in all previous simulations. For the Si sample, this saturation effect starts below 0.4 nm on the *x*-axis. On the other hand, the simulation times increase enormously with x values below 0.2 nm (h-BN sample) and 0.1 nm (Si sample). The final results of the present work were obtained by performing 60 single simulations, excluding the significantly larger number of “pre-simulations”. Therefore, the chosen meshing strategy with a maximum size of a triangular mesh element at the TCA of 0.2 nm provides a good compromise between accuracy and simulation time.

## 5. Conclusions

This work provides detailed insights into the heat transfer process during realistic SThM measurements. An SEM investigation of a used SThM probe made it possible to model an “average” used probe and calculate realistic values for the TCAs. Realistic values for thermal penetration depths, TARs, and NTEFs through the tip in contact with an h-BN or a Si sample, respectively, are provided. This allows other researchers to estimate said values for their special measurement setup and may help to interpret practical SThM measurements correctly or to explain occurring effects such as topography influences. In addition, the presented values for the TARs may help to evaluate the lateral resolution of SThM measurements as the TARs help to interpret the effect of adjacent layers. Similar to the proposal of other researchers [22], it could be shown that the influence of convection and radiation may be neglected in such studies.

From the authors’ point of view, one of the most interesting findings of this study is the great impact of possible TIRs, which may not be neglected. This work may also justify higher tip velocities in practical SThM measurements as t_stat_ is estimated below 1 µs. As a single SThM measurement can take more than 1 h, a possible increase of the tip velocities may accelerate practical measurements without a loss of thermal accuracy. However, t_stat_ only represents the stationary time of the heat propagation of the tip-sample contact. The time constant of the entire probe may slow down the sensing mechanism. In recent practical measurements, we tried to use higher tip velocities and compared the thermal images to a tip velocity of 1 µms^−1^ without significant differences. Said practical observation, therefore, agrees with the theoretical findings in this work and may justify tip velocities of up to 20 µms^−1^. Nevertheless, the values obtained in this work are only theoretical results, which could hardly be verified as almost no comparative results can be found in the literature so far. In the future, similar practical measurements need to be performed to verify the presented values.

## Figures and Tables

**Table 1:** Material properties are used for the theoretical calculation of the thermal contact areas (TCAs) in Figure 3. Values for SiO_2_ are approximated from the predefined material in COMSOL Multiphysics (CM).

Material	Si	SiO_2_	h-BN
Young’s modulus (GPa)	131 [26]	70	850 [28]
Poisson’s ratio (–)	0.221 [26]	0.157	0.2 [29]

## Data Availability

The data presented in this study are available on request from the corresponding author. The data are not publicly available due to the large amount of simulation data and the difficulty to present native modeling and simulation files in a reasonable way.

## References

[1] Cahill D.G., Goodson K., Majumdar A. (2002). Thermometry and Thermal Transport in Micro/Nanoscale Solid-State Devices and Structures. J. Heat Transf..

[2] Cahill D.G., Ford W.K., Goodson K.E., Mahan G.D., Majumdar A., Maris H.J., Merlin R., Phillpot S.R. (2003). Nanoscale thermal transport. J. Appl. Phys..

[3] Choi S.R., Kim D., Choa S.-H., Lee S.-H., Kim J.-K. (2006). Thermal Conductivity of AlN and SiC Thin Films. Int. J. Thermophys.

[4] Cahill D.G., Braun P.V., Chen G., Clarke D.R., Fan S., Goodson K.E., Keblinski P., King W.P., Mahan G.D., Majumdar A. (2014). Nanoscale thermal transport. II. 2003–2012. Appl. Phys. Rev..

[5] Price D.M., Reading M., Hammiche A., Pollock H.M. (1999). Micro-thermal analysis: Scanning thermal microscopy and localised thermal analysis. Int. J. Pharm..

[6] Ruiz F., Sun W.D., Pollak F.H., Venkatraman C. (1998). Determination of the thermal conductivity of diamond-like nanocomposite films using a scanning thermal microscope. Appl. Phys. Lett..

[7] Sadeghi M.M., Park S., Huang Y., Akinwande D., Yao Z., Murthy J., Shi L. (2016). Quantitative scanning thermal microscopy of graphene devices on flexible polyimide substrates. J. Appl. Phys..

[8] Martinek J., Klapetek P., Campbell A.C. (2015). Methods for topography artifacts compensation in scanning thermal microscopy. Ultramicroscopy.

[9] Shi L., Plyasunov S., Bachtold A., McEuen P.L., Majumdar A. (2000). Scanning thermal microscopy of carbon nanotubes using batch-fabricated probes. Appl. Phys. Lett..

[10] Hammiche A., Pollock H.M., Song M., Hourston D.J. (1996). Sub-surface imaging by scanning thermal microscopy. Meas. Sci. Technol..

[11] Majumdar A. (1999). SCANNING THERMAL MICROSCOPY. Annu. Rev. Mater. Sci..

[12] Metzke C., Frammelsberger W., Weber J., Kühnel F., Zhu K., Lanza M., Benstetter A.G. (2020). On the Limits of Scanning Thermal Microscopy of Ultrathin Films. Materials.

[13] Leitgeb V., Fladischer K., Mitterhuber L., Defregger S. Quantitative SThM Characterization for Heat Dissipation Through Thin Layers. Proceedings of the 2019 25th International Workshop on Thermal Investigations of ICs and Systems (THERMINIC).

[14] Fladischer K., Leitgeb V., Mitterhuber L., Maier G.A., Keckes J., Sagmeister M., Carniello S., Defregger S. (2019). Combined thermo-physical investigations of thin layers with Time Domain Thermoreflectance and Scanning Thermal Microscopy on the example of 500 nm thin, CVD grown tungsten. Thermochim. Acta.

[15] Chen W., Feng Y., Qiu L., Zhang X. (2020). Scanning thermal microscopy method for thermal conductivity measurement of a single SiO2 nanoparticle. Int. J. Heat Mass Transf..

[16] Park J., Koo S., Kim K. (2019). Measurement of thermal boundary resistance in ∼10 nm contact using UHV-SThM. IJNT.

[17] Chirtoc M., Bodzenta J., Kaźmierczak-Bałata A. (2020). Calibration of conductance channels and heat flux sharing in scanning thermal microscopy combining resistive thermal probes and pyroelectric sensors. Int. J. Heat Mass Transf..

[18] Nguyen T.P., Thiery L., Euphrasie S., Lemaire E., Khan S., Briand D., Aigouy L., Gomes S., Vairac P. (2019). Calibration Tools for Scanning Thermal Microscopy Probes Used in Temperature Measurement Mode. J. Heat Transf..

[19] Bruker VITA—NanoTA. https://www.brukerafmprobes.com/c-206-vita-nanota.aspx.

[20] Bruker VITA-DM-NANO-TA-200. https://www.brukerafmprobes.com/p-3701-vita-dm-nanota-200.aspx.

[21] Born A. (2000). Nanotechnologische Anwendungen der Rasterkapazitätsmikroskopie und Verwandter Rastersondenmethoden. Ph.D. Thesis.

[22] Zhang Y., Zhu W., Hui F., Lanza M., Borca-Tasciuc T., Muñoz Rojo M. (2019). A Review on Principles and Applications of Scanning Thermal Microscopy (SThM). Adv. Funct. Mater..

[23] Hering E., Modler K.-H. (2007). Grundwissen des Ingenieurs.

[24] Popov V.L. (2009). Rigorose Behandlung des Kontaktproblems–Hertzscher Kontakt. Kontaktmechanik und Reibung.

[25] Nitsche K., Marek R. (2007). Praxis der Wärmeübertragung. Grundlagen, Anwendungen, Übungsaufgaben; mit 48 Tabellen, 50 vollständig durchgerechneten Beispielen sowie 93 Übungsaufgaben mit Lösungen.

[26] Korth Kristalle GmbH Silizium (Si). https://www.korth.de/index.php/material-detailansicht/items/32.html.

[27] Frühauf J. (2005). Shape and Functional Elements of the Bulk Silicon Microtechnique. A Manual of Wet-Etched Silicon Structures.

[28] Falin A., Cai Q., Santos E.J.G., Scullion D., Qian D., Zhang R., Yang Z., Huang S., Watanabe K., Taniguchi T. (2017). Mechanical properties of atomically thin boron nitride and the role of interlayer interactions. Nat. Commun..

[29] Boldrin L., Scarpa F., Chowdhury R., Adhikari S. (2011). Effective mechanical properties of hexagonal boron nitride nanosheets. Nanotechnology.

[30] Frammelsberger W., Benstetter G., Kiely J., Stamp R. (2007). C-AFM-based thickness determination of thin and ultra-thin SiO 2 films by use of different conductive-coated probe tips. Appl. Surf. Sci..

[31] Jiang L., Shi Y., Hui F., Tang K., Wu Q., Pan C., Jing X., Uppal H., Palumbo F., Lu G. (2017). Dielectric Breakdown in Chemical Vapor Deposited Hexagonal Boron Nitride. ACS Appl. Mater. Interfaces.

[32] Villaroman D., Wang X., Dai W., Gan L., Wu R., Luo Z., Huang B. (2017). Interfacial thermal resistance across graphene/Al2O3 and graphene/metal interfaces and post-annealing effects. Carbon.

[33] Hong Y., Zhang J., Zeng X.C. (2016). Thermal contact resistance across a linear heterojunction within a hybrid graphene/hexagonal boron nitride sheet. Phys. Chem. Chem. Phys..

[34] Zhang J., Wang Y., Wang X. (2013). Rough contact is not always bad for interfacial energy coupling. Nanoscale.

[35] Timofeeva M., Bolshakov A., Tovee P.D., Zeze D.A., Dubrovskii V.G., Kolosov O.V. (2013). Nanoscale Resolution Scanning Thermal Microscopy with Thermally Conductive Nanowire Probes. arXiv.

[36] Kim K., Jeong W., Lee W., Sadat S., Thompson D., Meyhofer E., Reddy P. (2014). Quantification of thermal and contact resistances of scanning thermal probes. Appl. Phys. Lett..

[37] Timofeeva M., Bolshakov A., Tovee P.D., Zeze D.A., Dubrovskii V.G., Kolosov O.V. (2016). Scanning thermal microscopy with heat conductive nanowire probes. Ultramicroscopy.

[38] Song Y., Mandelli D., Hod O., Urbakh M., Ma M., Zheng Q. (2018). Robust microscale superlubricity in graphite/hexagonal boron nitride layered heterojunctions. Nat. Mater..

[39] Hwang G., Chung J., Kwon O. (2014). Enabling low-noise null-point scanning thermal microscopy by the optimization of scanning thermal microscope probe through a rigorous theory of quantitative measurement. Rev. Sci. Instrum..

[40] Assy A., Gomès S. (2015). Heat transfer at nanoscale contacts investigated with scanning thermal microscopy. Appl. Phys. Lett..

[41] Ravindra N.M., Sopori B., Gokce O.H., Cheng S.X., Shenoy A., Jin L., Abedrabbo S., Chen W., Zhang Y. (2001). Emissivity Measurements and Modeling of Silicon-Related Materials: An Overview. Int. J. Thermophys.

[42] Cerbe G., Hoffmann H.-J. (1990). Einführung in die Wärmelehre. Von der Thermodynamik zur technischen Anwendung; mit 30 Tafeln, 111 Beispielen, 119 Aufgaben und 161 Kontrollfragen.

